# A novel stress-based intervention reduces cigarette use in non-treatment seeking smokers

**DOI:** 10.1038/s41386-022-01455-6

**Published:** 2022-09-29

**Authors:** Alexandra Barnabe, Karine Gamache, João Vitor Paes de Camargo, Erin Allen-Flanagan, Mathilde Rioux, Jens Pruessner, Marco Leyton, Karim Nader

**Affiliations:** 1 Department of Psychology, McGill University, Montreal, QC Canada; 2 Department of Psychology, University of Konstanz, Baden-Württemberg, Germany; 3 Department of Psychiatry, McGill University, Montreal, QC Canada; 4 Department of Neurology & Neurosurgery, Montreal Neurological Institute, McGill University, Montreal, QC Canada; 5 Center for Studies in Behavioral Neurobiology, Concordia University, Montreal, QC Canada

**Keywords:** Addiction, Human behaviour

## Abstract

Tobacco use is the leading cause of preventable mortality worldwide. Since current smoking cessation aids show only modest efficacy, new interventions are needed. Given the evidence that stress is a potent trigger for smoking, the present randomized clinical trial tested whether stress could augment the effects of a memory updating (retrieval-extinction) intervention. Non-treatment seeking smokers (*n* = 76) were assigned to one of four conditions composed of either a stressful or non-stressful psychosocial challenge followed by either smoking or neutral cues. Ten minutes after this manipulation, all underwent a 60-minute extinction procedure during which they viewed smoking-related videos and images and manipulated smoking paraphernalia. Compared to participants who were not exposed to the laboratory stressor, the stressor-exposed groups exhibited greater psychophysiological responses during their intervention and greater decreases in cigarette use at two- and six-weeks follow-up independent of smoking cue exposure. Together, these findings suggest that the ability of stress to activate cigarette seeking processes can be exploited to decrease cigarette use. With replication, the stress-based intervention could become a novel strategy for decreasing cigarette use in non-treatment seeking smokers.

Clinicaltrials.gov identifier: NCT04843969.

## Introduction

Tobacco use is the primary cause of preventable death worldwide [1]. While most tobacco users are familiar with the consequences of smoking, abstinence remains challenging for many. Among the most commonly reported barriers are the perceived difficulties of quitting and coping with stress [2–4]. Cessation aids are available but are infrequently used [5] owing partly to their modest efficacy [6–9] and high cost [10, 11]. Together, these observations highlight the need for new treatments that can increase motivation to quit and improve remission rates.

A fundamental feature of smoking is the learned associations between smoking behaviors and cigarette-related cues [12]. Exposure to these cues can potently trigger both craving and relapse [13–20]. One potential treatment approach involves weakening the strength of these associations, such as through memory reconsolidation blockade. Memory reconsolidation theory postulates that stable (consolidated) memories become labile when recalled and are thus susceptible to modification. While in this labile state, it is possible to interfere with the restabilization (reconsolidation), a process known as memory reconsolidation blockade.

In the past two decades, memory reconsolidation blockade interventions have shown promise as treatments for stress and anxiety related disorders [21–25]. These interventions have typically used pharmacological agents to impair reconsolidation and weaken memory associations. More recent work has tested the potential of less invasive behavioral approaches. One such intervention, known as memory updating, involves three distinct phases: (1) a reactivation phase during which a brief reminder triggers the recall of target memories and renders them labile, vulnerable to modification; (2) a short interval without memory-eliciting stimuli; and (3) an extinction phase during which a lengthy cue exposure alters the elicited memories by disrupting their reconsolidation (restabilization), thereby reducing their potency [26].

Memory updating might also be applied to substance use problems by weakening the ability of drug-associated triggers to elicit substance use [27–29]. In recently abstinent heroin-addicted inpatients, two sessions of a memory updating intervention using drug-related cues decreased craving responses during re-exposure to the same cues [29]. A similar approach reduced both cravings and cigarette use in treatment-seeking smokers [27].

In the present study, we aimed to extend the previous findings and further decrease cigarette smoking using a novel procedure. Given the evidence that stress can potently trigger cigarette cravings [30, 31] and use [2–4, 32], and increase the retrieval of other drug-related memories [33], we hypothesized that a stress task could augment the effects of a memory updating procedure. To test this, we compared four different conditions (phase 1) as part of a single-session intervention: (1) a stress task followed by smoking cues, (2) a stress task followed by neutral cues, (3) a non-stressful task followed by smoking cues, and (4) a non-stressful task followed by neutral cues. Following a 10-minute period without smoking cues or stress task, participants in all four conditions underwent a 60-minute exposure to smoking-related cues (phase 2). Adding to the study’s novelty, all participants were non-treatment seeking.

Based on the literature described above, we made two main predictions. First, compared to all other groups, the combined stress and cue procedure would induce greater craving and physiological responses during phase 1 and larger decreases in cue reactivity and cigarette use at two- and six-week follow-up. Second, the stress-based intervention alone would be at least as effective as the cue-based intervention alone.

## Methods

### Participants

Non-treatment seeking smokers were recruited through online advertisements, flyers posted around Montreal, and word-of-mouth. Study eligibility was determined from telephone interviews using the Fagerström Test for Cigarette Dependence (FTCD) [34, 35] and the Mini International Neuropsychiatric Interview [36, 37]. Primary inclusion criteria included scoring 5 or higher on the FTCD, willingness to abstain from smoking for four hours prior to each laboratory visit and being between 18 and 65 years of age. Exclusion criteria included current use of cigarette cessation products, ß-blockers, antidepressant, anxiolytic or other psychotropic medications, pregnancy, and meeting diagnostic criteria for current (untreated) psychological disorders (see Supplementary Methods for additional details). The study was carried out in accordance with the Declaration of Helsinki and approved by the McGill University Research Ethics Board. All participants were informed that study participation could affect their smoking habits and cravings prior to providing written informed consent.

### Procedures

**Fig. 1:**
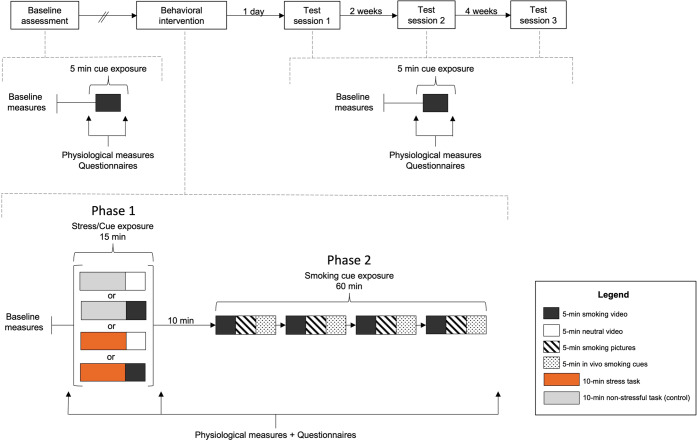
Experimental design and timeline. During the initial assessment and test sessions 1, 2 and 3, a novel smoking-related video was shown to participants. Physiological measures (heart rate (HR) and skin conductance (SC)) were collected prior to and during the last minute of each video presentation. During these same visits, blood pressure (BP) and self-reported cigarette craving and urge to smoke scores were collected pre- and post-video. On the day of the behavioral intervention, baseline physiological measures (HR, SC, and BP) and self-report data were gathered, followed by one of four conditions (phase 1): stress task and smoking cue, stress task and neutral cue, non-stressful task and smoking cue, or non-stressful task and neutral cue. Physiological and craving measures were collected and followed by a 10-minute break. All participants then went through the extinction protocol (phase 2) consisting of additional smoking videos, smoking images, and the manipulation of smoking-related paraphernalia. Immediately after phase 2, physiological and craving measures were gathered again.

This randomized clinical trial took place between February 2019 and January 2020. The study comprised five in-person visits, including baseline, intervention, and three test sessions given 24 h, two weeks and six weeks post-intervention (Fig. 1). All visits were standardized and administered by the same experimenter (AB). Participants were asked to remain abstinent for four hours prior to each visit. To promote compliance, breath carbon monoxide (CO) measures were collected at the start of the session, but they were not analyzed as the breath monitor lacks sensitivity to detect short periods of abstinence [38, 39].

#### Baseline

During the first study visit, participants provided demographic information, including age, sex, ethnicity, level of education, employment status, and history of cigarette use. They completed the Contemplation Ladder [40] to evaluate their readiness to quit smoking and the Cigarette Withdrawal Scale (CWS-21) [41] to assess symptoms of withdrawal on six dimensions: craving, insomnia, depression–anxiety, appetite–weight gain, irritability–impatience, and difficulty concentrating. They then had a baseline cue reactivity assessment during which they viewed a five-minute video containing ten 30-second smoking-related clips (see Supplementary Methods). Pre- and post-video conscious craving responses were measured using the Tobacco Craving Questionnaire––Short Form (TCQ-SF) [42] and the Questionnaire on Smoking Urges––Brief (QSU-Brief) [43]. Physiological measures, including heart rate (HR) and skin conductance (SC), were recorded for one minute pre-video and during the last minute of the five-minute video cue using the ProComp Infinity 5-channel, multi-modality encoder (Thought Technology Ltd, Montreal, Canada). Blood pressure (BP) was collected pre- and post-video cue presentation.

#### Intervention

Prior to the baseline session, participants were randomized to one of four conditions (phase 1) using a 2 × 2 factorial design: (1) stress task and smoking cue, (2) stress task and neutral cue, (3) control task and smoking cue, or (4) control task and neutral cue. The stress task was the Montreal Imaging Stress Test (MIST) [44], a validated psychosocial challenge consisting of competitive mental arithmetic combined with negative social evaluation (see Supplementary Methods). Participants were blind to group allocation and study hypotheses.

The MIST (or its control version) was immediately followed by a five-minute video cue presentation. For participants in the smoking cue condition, these video clips were similar but non-identical to those presented at baseline. For participants in the neutral cue condition, cues consisted of ten 30-second clips depicting non-smoking activities (see Supplementary Methods). Neutral and smoking cues were matched on number of people in each clip, their approximate age, ethnicity, distance from the camera, and lighting.

All four phase 1 conditions were followed by a 10-minute break during which participants remained seated in front of a black screen. They then underwent a 60-minute extinction protocol (phase 2). This entailed four rotations of: a five-minute video with smoking-related content (composed of similar but non-identical clips to those presented in the baseline visit), a five-minute presentation of smoking images (with each image presented for 3 seconds, see Supplementary Methods), and five minutes of manipulating smoking paraphernalia (e.g., lighter, cigarettes).

Immediately prior to and after phase 1, and immediately after phase 2, HR, SC and BP were measured, and participants completed the TCQ-SF and QSU-Brief. Participants remained in the laboratory for one hour following completion of the questionnaires to minimize the chance that they would reengage the association between cigarette use and smoking-related cues. Participants who underwent the stressful MIST were debriefed. All participants were asked to see how long they could go without smoking after the session.

#### Test sessions 1, 2 and 3

Participants returned to the laboratory for cue reactivity test sessions 24 h, two weeks, and six weeks following the intervention. During each of these sessions, SC, HR and BP were measured, and the CWS-21, Contemplation Ladder, FTCD (for tests 2 and 3), TCQ-SF and QSU-Brief were administered. Participants then viewed a new five-minute smoking cue video with SC and HR measured for one minute before video presentation and again during the last minute of the video. Immediately after the video, BP was measured and the TCQ-SF and QSU-Brief were administered again.

Between each of the test sessions, participants were asked to record their daily cigarette use in a journal provided by the experimenters. Data from the journals were collected at test sessions 2 and 3.

### Statistical analyses

SPSS 26.0.0.1 (Chicago, IL) was used for all statistical analyses. Preliminary analyses indicated less than 1% missing data on all variables. Multiple imputation was used to impute missing scores. Chi square tests and analyses of variance (ANOVAs) were used to examine group differences in study characteristics at baseline for categorical and continuous variables, respectively. Daily cigarette use at test sessions 2 and 3 was calculated as the mean number of cigarettes smoked per day in the week prior to each test session. Repeated measures analyses of variance (ANOVA) were used to examine group differences in physiological and craving measures, daily cigarette use, Contemplation Ladder and FTCD scores. Partial eta squared were used to assess the magnitude of these effects. Greenhouse-Geisser corrections were applied when the assumption of sphericity was violated. Post-hoc analyses consisted of paired samples t-tests with Bonferroni corrections. Correlational analyses tested for potential predictors of cigarette use changes, and the Benjamini-Hochberg procedure with a false discovery rate of 5% was applied to decrease the risk of false positives.

## Results

### Participant characteristics

**Fig. 2:**
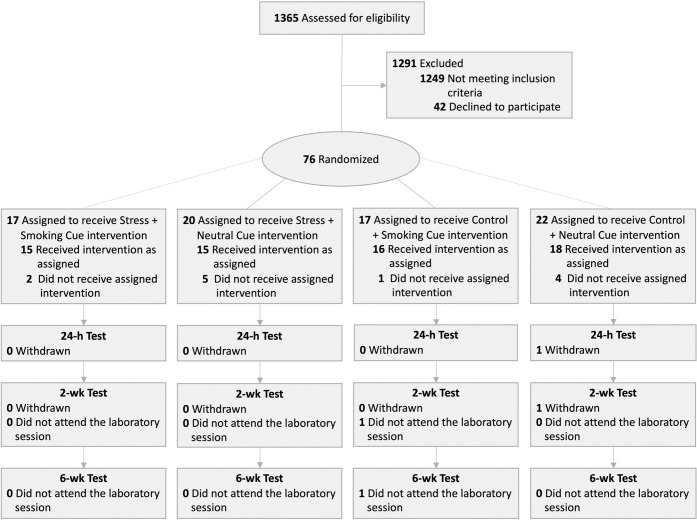
CONSORT Flow Diagram. Reasons for withdrawal after randomization included reduction or cessation in cigarette use prior to the intervention, misreporting cigarette use, and a participant having dyscalculia. Scheduling conflicts or time constraints were the primary reasons why some participants declined to participate.

Seventy-six volunteers were deemed eligible for the study (Fig. 2). Of these, 14 withdrew or were withdrawn. Reasons for withdrawal included dyscalculia, scheduling conflicts, and not meeting study requirements. Of the remaining 62 participants, one did not attend test session 2 for medical reasons and another missed test session 3 due to relocating to another province. When the participants were randomly assigned to the subgroups, there were no significant differences in tobacco use (16.9 ± 5.9 cigarettes per day; *n* = 62) or other demographic or clinical characteristics (Table 1).

**Table 1:** Demographic and Clinical Characteristics of Treatment Groups^a^.

		Participants, No. (%)/GROUP	CONTROL		STRESS			
Characteristic		All participants (*n* = 62)	Control stressor + Neutral Cues (*n* = 16)	Control stressor + Smoking Cues (*n* = 16)	Stressful task + Neutral Cues (*n* = 15)	Stressful task + Smoking cues (*n* = 15)	Statistic	*P* Value
Age, mean (SD)		35.82 (12.99)	38.56 (13.45)	38.25 (14.85)	31.47 (10.92)	34.67 (12.18)	*F* = 1.03	0.39
Sex								
	Male	34 (54.84)	9 (56.25)	9 (56.25)	8 (53.33)	8 (53.33)	χ2 = 0.05	1.00
	Female	28 (45.16)	7 (43.75)	7 (43.75)	7 (46.67)	7 (46.67)		
Ethnicity								
	White	45 (72.58)	13 (81.25)	10 (62.50)	12 (80.00)	10 (66.67)	χ2 = 2.10	0.55
	Other^b^	17 (27.42)	3 (18.75)	6 (37.50)	3 (20.00)	5 (33.33)		
Employed								
	Yes	39 (62.90)	10 (62.50)	8 (50.00)	11 (73.33)	10 (66.67)	χ2 = 1.93	0.59
	No	23 (37.10)	6 (37.50)	8 (50.00)	4 (26.67)	5 (33.33)		
Education								
	No HS completion	2 (3.23)	1 (6.25)	1 (6.25)	0 (0)	0 (0)	χ2 = 2.94	0.97
	HS graduate	9 (14.52)	2 (12.50)	2 (12.50)	3 (20.00)	2 (13.33)		
	College or trade school graduate	21 (33.87)	6 (37.50)	6 (37.50)	4 (26.67)	5 (33.33)		
	University graduate	30 (48.39)	7 (43.75)	7 (43.75)	8 (53.33)	8 (53.33)		
Annual household income, $								
	≤20,000	21 (33.87)	3 (18.75)	6 (37.50)	8 (53.33)	4 (26.67)	χ2 = 4.61	0.20
	>20,000	41 (66.13)	13 (81.25)	10 (62.50)	7 (46.67)	11 (73.33)		
No. of cigarettes smoked per day, mean (SD)		16.87 (5.87)	16.84 (6.17)	15.34 (5.71)	18.27 (6.64)	17.13 (5.07)	*F* = 0.64	0.59
CO level (ppm), mean (SD)		8.60 (5.72)	11.25 (5.92)	6.75 (5.47)	7.67 (7.08)	8.67 (3.18)	*F* = 1.92	0.14
FTCD score, mean (SD)		6.05 (1.19)	5.81 (1.05)	6.13 (1.02)	6.00 (1.36)	6.27 (1.39)	*F* = 0.39	0.76
Contemplation Ladder score, mean (SD)		4.97 (1.59)	4.75 (1.34)	5.13 (1.78)	4.87 (1.55)	5.13 (1.77)	*F* = 0.22	0.88
Age at first cigarette smoked, mean (SD)		16.08 (4.14)	15.91 (2.95)	16.56 (3.31)	14.87 (3.74)	16.97 (6.08)	*F* = 0.73	0.54
Years of smoking, mean (SD)		19.74 (14.07)	22.66 (15.06)	21.69 (15.43)	16.60 (12.86)	17.70 (13.04)	*F* = 0.67	0.57
No. of MINI diagnoses								
	0	52 (83.9)	14 (87.50)	14 (87.50)	11 (73.33)	13 (86.67)	χ2 = 1.63	0.65
	1	10 (16.1)	2 (12.50)	2 (12.50)	4 (26.67)	2 (13.33)		

*CO* carbon monoxide, *FTCD* Fagerström Test for Cigarette Dependence, *HS* high school, *MINI* Mini International Neuropsychiatric Interview.

^a^All measures were collected at baseline.

^b^Other self-reported ethnicities included African American, Asian, Hispanic/Latinx, Indian, Indigenous, Middle Eastern, and other.

### Experimental manipulation check

Since this was the first time stress and smoking cues were combined in a memory updating procedure, we initially examined whether they elicited their expected effects during phase 1 of the intervention session. As hypothesized, the stress task yielded the predicted effects within the smoking cue groups (*P*s < 0.05 for SC, systolic BP and QSU-Brief scores) and the neutral cue groups (*P*s < 0.05 for TCQ-SF and QSU-Brief scores). In contrast, the smoking cues, administered immediately after the stress and non-stress tasks, did not change either craving or physiological responses, not within the stress task groups (all *P*s > 0.30) or the non-stressful task groups (all *P*s > 0.10), nor were there any stress by cue interactions (all *P*s > 0.30) (Supplementary Figs. 3 and 5). Based on these observations and an absence of cue effects on cigarette use at follow-up (all *P*s > 0.25) (Supplementary Fig. 4), subsequent analyses combined the two stress subgroups (stress) and two non-stressful subgroups (controls), thus increasing statistical power to detect effects of stress.

### Responses during the intervention

#### Phase 1

**Fig. 3:**
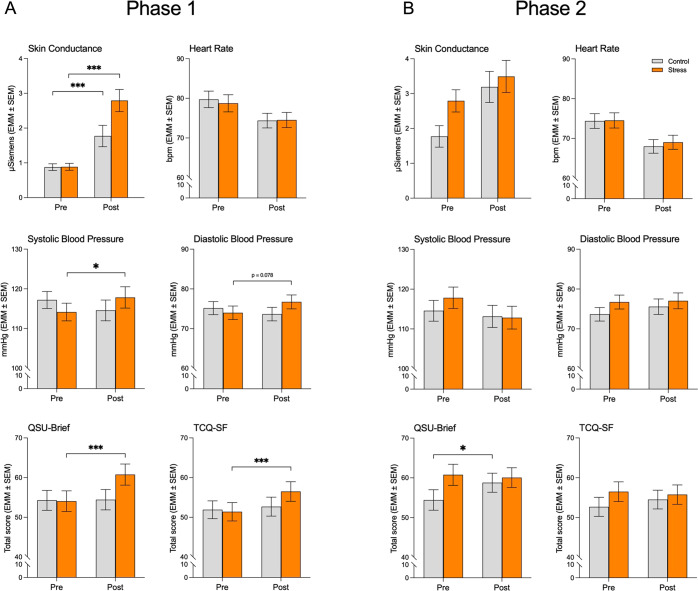
The stress task-induced psychophysiological and craving responses during the behavioral intervention. During phase 1 (**A**), participants exposed to the stress task exhibited larger increases in physiological (SC and systolic BP) and craving measures (QSU-Brief and TCQ-SF scores). During phase 2 (**B**), physiological measures did not significantly differ between groups, but participants previously exposed to the control task exhibited greater smoking cue-induced craving responses (QSU-Brief). BP: blood pressure; SC: skin conductance; TCQ-SF: Tobacco Craving Questionnaire – Short Form; QSU-Brief: Questionnaire on Smoking Urges – Brief. EMM: estimated marginal mean; SEM standard error of the mean. *P < 0.05, ***P < 0.001.

When comparing scores obtained immediately pre- and post-phase 1, there were main effects of time reflecting anticipated decreases in HR (*F*_1,60_ = 61.61, *P* < 0.001) and increases in SC (*F*_1,60_ = 63.04, *P* < 0.001) and craving (QSU-Brief: *F*_1,60_ = 14.90, *P* < 0.001; TCQ-SF: *F*_1,60_ = 14.32, *P* < 0.001). The increases in physiological and craving scores were larger in the stress group, compared to the controls (Fig. 3A), as reflected by stress condition by time interactions for SC (*F*_1,60_ = 8.22, *P* = 0.006), BP (systolic: *F*_1,60_ = 8.65, *P* = 0.005; diastolic: *F*_1,60_ = 5.52, *P* = 0.022), and craving (QSU-Brief: *F*_1,60_ = 13.58, *P* < 0.001; TCQ-SF: *F*_1,60_ = 7.72, *P* = 0.007). There were no group differences for HR.

#### Phase 2

Across groups, the extinction procedure significantly increased SC (*F*_1,60_ = 29.60, *P* < 0.001) and decreased both HR (*F*_1,60_ = 68.94, *P* < 0.001) and systolic BP (*F*_1,60_ = 5.85, *P* = 0.019). The stress and control groups were not significantly different on most measures but there was a stress condition by time interaction for QSU-Brief (*F*_1,60_ = 5.03, *P* = 0.029) reflecting a significant increase in craving during phase 2 only in the control group (*P* = 0.014) (Fig. 3B).

### Cue reactivity assessments

#### Baseline

As expected, exposure to a smoking-related video led to a main effect of time revealing significant increases in craving (QSU-Brief: *F*_1,60_ = 15.01, *P* < 0.001; TCQ-SF: *F*_1,60_ = 13.96, *P* < 0.001) and SC responses (*F*_1,60_ = 46.67, *P* < 0.001) and decreases in HR (*F*_1,60_ = 39.11, *P* < 0.001) and systolic BP responses (*F*_1,60_ = 4.20, *P* = 0.045). There was a single stress condition by time interaction (systolic BP: *F*_1,60_ = 11.76, *P* = 0.001; Fig. S1C), which was likely spurious given that groups were not yet treated differently. This interaction was controlled for in subsequent analyses.

#### Tests 1, 2 and 3

Across groups, at each test session, exposure to a new smoking-related video led to increased SC response (all *P*s < 0.001) and decreased HR (all *Ps* < 0.002). Increases in craving were seen at test sessions 1 (TCQ-SF: *P* < 0.05) and 2 (QSU-Brief: *P* < 0.001) but not at test session 3. See Supplementary Results and Supplementary Fig. 1 for additional details.

#### Across sessions

Across sessions, physiological responses to the video cues became less pronounced as shown by decreases in SC ((*F*_3,180_ = 5.52, *P* = 0.001) from baseline to test 1 (*P* = 0.008) and baseline to test 3 (*P* = 0.031)) and increases in HR ((*F*_3,180_ = 2.90, *P* = 0.036) from baseline to test 3 (*P* = 0.033)). No across session changes or group differences in craving were observed. See Supplementary Results and Supplementary Fig. 1 for additional details.

### Smoking behavior

**Fig. 4:**
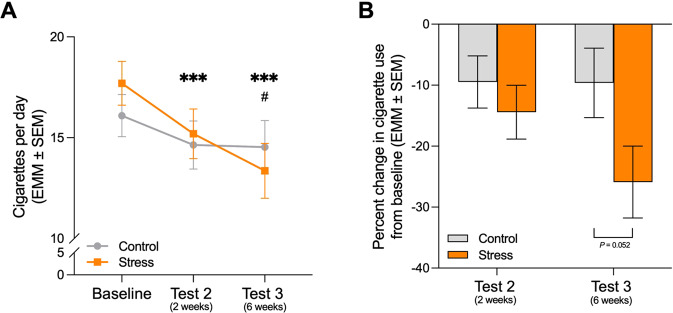
The stress-based memory intervention led to decreased cigarette use at follow-up. **A** Participants in the stress-based intervention significantly decreased their average daily cigarette use from baseline to the tests 2 and 3 (two and six weeks after the intervention, respectively). **B** Cigarette use in the stress group participants decreased by 14% at two weeks (test 2) and by 26% at six weeks (test 3), while the control group showed decreases of 9% and 10%, respectively. EMM: estimated marginal mean; SEM: standard error of the mean. In the stress group: ***P ≤ 0.001 compared to baseline, #*P* < 0.05 compared to test 2.

Over the period of the study, participants decreased their cigarette use as reflected by a significant main effect of session (*F*_1.66,99.35_ = 16.58, *P* < 0.001). Stress condition by session interactions were observed for baseline vs. test sessions 2 and 3 (*F*_1.66,99.35_ = 3.60, *P* = 0.039, η_p_^2^ = 0.057) and baseline vs. test session 3 alone (*F*_1,60_ = 4.86, *P* = 0.031, η_p_^2^ = 0.075) (Fig. 4A). These interactions reflected significant decreases in cigarette smoking in the stress group participants from baseline to test session 2 (*P* = 0.001) and baseline to test session 3 (*P* < 0.001) but not in the control group participants (all *P*s > 0.05). This represented decreases of 14% and 26% in the stress group compared to 9% and 10% in the controls at test sessions 2 (*F*_1,62_ = 0.67, *P* = 0.42) and 3 (*F*_1,62_ = 3.94, *P* = 0.052), respectively (Fig. 4B). The magnitude of the intervention’s effect increased over time such that, in the stress group, cigarettes per day decreased from baseline and were lower at test session 3 compared to test 2 (*P* = 0.020). No significant changes in cigarette use were found in the controls.

Across study visits, there were progressive decreases in cigarette dependence (FTCD: *F*_2,120_ = 10.94, *P* < 0.001) and withdrawal symptoms due to craving (CWS-21 craving subscale: *F*_3,180_ = 8.05, *P* < 0.001), and increased motivation to quit smoking scores (Contemplation Ladder: *F*_2.46,147.93_ = 9.86, *P* < 0.001) (Supplementary Table 1). There were no significant group differences in these measures and no main effects of session on other CWS-21 subscales.

### Predictors of decreased cigarette use

When participants from all groups were combined, higher systolic BP values following phase 1 of the intervention predicted larger decreases in cigarette use at test session 3 (*r* = −0.272, *P* = 0.033) (Supplementary Fig. 2A). Furthermore, larger decreases in systolic BP during phase 2 predicted greater decreases in cigarette use at test session 2 (*r* = 0.275, *P* = 0.030; Supplementary Fig. 2B) and test session 3 (*r* = 0.379, *P* = 0.002; Supplementary Fig. 2C). This latter result survived the Benjamini-Hochberg correction.

## Discussion

The present study tested whether a laboratory stressor could augment the ability of a memory updating procedure to decrease cigarette use. While our predictions for the cue-based intervention were not supported, two hypotheses about the stressor were. First, during phase 1 of the intervention, greater craving and physiological responses were observed following exposure to the stressor as compared to the control task. Second, and more importantly, during the weeks following the intervention, larger decreases in cigarette use were seen in stress versus control group participants.

The failure to reproduce the effect of a previous smoking cue-based intervention on cigarette use [27] might be related to the inability of the present study’s smoking cues to elicit psychophysiological responses when they were combined with the arithmetic tasks (both stress and control versions). This was unexpected since our smoking cues yielded their anticipated effects [45, 46] when administered alone at the baseline and follow-up sessions. Several factors might explain this: (1) the arithmetic tasks may have served as distractors reducing the effectiveness of the smoking cues, (2) the stressful version of the task might have elicited near maximal responses that were not further augmented by exposure to the smoking cues, (3) habituation to the video cues from baseline to phase 1 could not be ruled out, but this was considered unlikely as novel videos were presented at each session and the expected cue-induced responses re-emerged during the follow-up sessions.

The stress-based intervention reduced cigarette use but not craving and physiological responses during the follow-up sessions. This too differed from studies using cue-based memory updating interventions [27, 29] and may reflect several procedural differences in addition to the features noted above. As a start, we used a single intervention session whereas the previous studies provided two sessions [27, 29]. Second, the minimum duration of abstinence prior to each session was four hours as compared to at least 24 hours in previous work [27, 29]. Third, we tested non-treatment seeking volunteers instead of those either seeking [27] or receiving treatment [27, 29]. Indeed, non-treatment seeking smokers exhibit larger smoking cue-reactivity responses than treatment-seekers [47], potentially accounting for the persisting responses across test sessions. Despite this continued ability of smoking cues to elicit craving, the stress-based intervention affected our primary outcome, cigarette use, which decreased by 26% in the stress group (as compared to 10% in controls) by six weeks. Dissociations between self-reported craving and substance use are frequently reported and are thought to indicate that drug-seeking behaviors are driven in large part by processes outside of conscious awareness [48–50]. The present study therefore suggests that the stress-based intervention may be more effective at targeting the preconscious processes. Further studies with longer follow-up periods will be needed to determine whether the behavioral changes persist, increase further, or eventually dissipate.

Interestingly, the control procedure also conferred some benefit. Across groups, we observed decreased nicotine dependence scores, increased motivation to quit smoking, and decreased cigarette use. While this may in part be a byproduct of study participation (e.g., placebo or Hawthorne effect), it may also reflect individual differences in responsiveness to the intervention. Just as the stress task may not have been equally stressful to all participants, for some, the control arithmetic task may have been stressful. These individual differences in stress reactivity may account for the correlations between physiological responding at intervention and changes in cigarette use. Across all participants, greater decreases in cigarette use correlated with greater phase 1 systolic BP responses and greater decreases in systolic BP during phase 2. Systolic BP responses could potentially reflect both the reactivation of craving-related processes and the efficacy of the intervention.

While this study was originally conceived as a memory updating paradigm, other interpretations are plausible. One possible interpretation is that phase 2 alone led to decreased cigarette use. However, although extinction procedures (often in the form of exposure therapy) can produce transient reductions in cigarette use [51, 52], long-term clinical efficacy is lacking [51–56]. More importantly, in the present study, all participants were exposed to a 60-minute extinction phase yet only those also exposed to the stressor exhibited significant decreases in cigarette use. It is therefore considered unlikely that extinction alone accounted for the present results.

A more plausible alternative interpretation may be that the stress procedure enhanced extinction. Although no studies (to our knowledge) have investigated behavioral stress induction on extinction (as in the current study), cortisol has been used to induce a stress state. In fear-related studies, elevated stress hormones during extinction learning (either endogenously or through prior cortisol administration) can further reduce fear responses post-extinction and at follow-up [57–62]. In the only study to date examining the role of pre-extinction stress on appetitive memories in a clinical population, administration of cortisol before exposure to alcohol cues decreased within-session cravings in those with a severe alcohol use disorder but increased cravings in those with less severe symptoms [63]. These limited results with a short follow-up (<8 days) are challenging to interpret and compare with the current findings. Here, as well as in cue-based memory updating studies [27, 64, 65], beneficial outcomes were evident at later time points but not during the extinction session or shortly after it. In contrast, many enhanced extinction studies report favorable effects during or soon after exposure to cues [57, 59, 66]. These different within-exposure session effects may indicate that different mechanisms can lead to similar outcomes, though with different time-courses.

While it is not possible to determine which mechanism (i.e., memory updating or enhanced extinction) is involved, the present study nonetheless provides, to our knowledge, the first evidence that a single-session stress-based intervention can reduce cigarette use in non-treatment seeking smokers. Future studies may wish to examine neurobiological underpinnings and clarify differences between enhanced extinction and memory updating.

## Supplementary information

Supplemental Material
