# Supplementary material for: A novel stress-based intervention reduces cigarette use in non-treatment seeking smokers
Source: Neuropsychopharmacology. 2022 Sep 29;48(2):308–16. doi: 10.1038/s41386-022-01455-6 (PMC9750979; doi:10.1038/s41386-022-01455-6)
Supplement: Supplementary file 1 — Supplemental Material [file 41386_2022_1455_MOESM1_ESM.docx]

**Supplemental Material**

**Supplementary Methods**

***Exclusion Criteria***

1. Moderate to severe psychiatric disorders: lifetime history of psychoses or current post-traumatic stress disorder, mood disorder, panic or other anxiety disorder.
2. Current treatment with ß-blockers or psychostimulants.
3. Pregnancy.

***Smoking and Neutral Cues***

Smoking-related videos were acquired from Dr. Joel Erblich’s research group at Mount Sinai School of Medicine, Dr. Edythe London’s research group at University of California, and YouTube. The YouTube cues were qualitatively similar to the previously validated Erblich and London lab videos [1,2]. Video cues were counterbalanced across the five sessions on the following criteria: number of smokers, their approximate age, sex, ethnicity, distance from camera, location, brightness, and video quality. Neutral videos were acquired from Dr. Erblich’s research group and similar video clip segments from YouTube.

Smoking-related images were acquired from the International Smoking Image Series, courtesy of Dr. Stephen Tiffany’s research group, SUNY, University at Buffalo [3], and from Dr. Charlotte Boettiger’s research group at University of North Carolina at Chapel Hill [4], Dr. Matt Field from the University of Sheffield [5], Dr. Maartje Luitjen’s research group at Radboud University [6], and Drs. Paul Pauli and Ronald Mucha at the University of Würzburg [7-9]. Pictures were categorized and counterbalanced across the four image sets on the following criteria: presence/absence of a person, their approximate age, sex, ethnicity, distance from camera, image quality and brightness.

***Montreal Imaging Stress Test***

During the MIST [10], participants were asked to solve computerized mental arithmetic problems while receiving negative social-evaluative feedback from both the experimenter and an on-screen performance progress bar. The arithmetic problems consisted of timed multiplication, division, addition and subtraction tasks which, unbeknownst to participants, were adjusted to their individual performance to enforce a 55% failure rate. On the computer monitor, participants received continuous social evaluation via a performance progress bar which indicated their performance in comparison to the (fictitious) average performance of other participants. They also received negative performance commentary from the experimenter between each run of the MIST. In the non-stressful control task of the MIST, participants performed similar arithmetic problems without time constraints or social evaluation. The experimenter provided neutral feedback, unrelated to the participant’s performance. All participants were exposed to three runs of the MIST, each lasting three minutes and followed by experimenter feedback. The MIST is well-validated as a laboratory stressor, previously shown to increase blood pressure [11], heart rate [11-13] and cortisol responses [11,13].

***Excluded Measures***

The following measures were collected but not included: Profile of Mood States – Short Form (POMS-SF) [14], Substance Use Risk Profile Scale (SURPS) [15], Mini-International Personality Item Pool (Mini-IPIP) [16], Perceived Stress Scale (PSS-10) [17], a lab-made checklist for the DSM-5 Criteria for Tobacco Use Disorder [18], and salivary alpha-amylase. Reasons for exclusion include problematic timing to test questions of interest (POMS-SF, PSS-10, alpha-amylase) and lack of association with the outcome measures (SURPS, Mini-IPIP). The analysis of telephone follow-ups six months post-intervention was omitted due to potential confounds (e.g., onset of COVID-19 and related changes in daily habits).

***Sample Size Calculation***

Based on an anticipated effect size between 0.4 and 0.5, sample sizes of 60 to 90 would yield power of 80%, 0.05 alpha error probability. Since power analyses tend to be conservative, we planned to test up to 80 participants. A first data check at N=62 confirmed that our objectives had been met.

***Randomization***

First author (AB) generated separate blocked randomization lists for males and females separately using an online random number generator [19] and assigned participants to intervention groups.

***Statistical Analyses***

Analyses of variance (ANOVAs) were used to examine group differences in study characteristics at baseline for categorical and continuous variables, respectively. Daily cigarette use at test sessions 2 and 3 was calculated as the mean number of cigarettes smoked per day in the week prior to each test session. Repeated measures analyses of variance were used to examine group differences in physiological and craving measures, daily cigarette use, Contemplation Ladder and FTCD scores. Greenhouse-Geisser corrections were applied when the assumption of sphericity was violated. Post-hoc analyses consisted of paired samples t-tests with Bonferroni corrections, unless otherwise specified.

**Supplementary Results**

***Cue Reactivity Assessments***

Following the intervention, participants underwent three test sessions:

***Test 1.*** Across groups, exposure to a new smoking-related video led to significantly increased craving (TCQ-SF: *F*_1,60_ = 5.88, *P* = 0.018; but not QSU-Brief: *P* > 0.05) and SC responses (*F*_1,60_ = 20.47, *P* < 0.001) and decreased HR (*F*_1,60_ = 24.02, *P* < 0.001) and BP responses (systolic: *F*_1,59_ = 7.71, *P* = 0.007; diastolic: *F*_1,60_ = 33.99, *P* < 0.001). There were no group differences in craving or physiological responses to the video at this session (Figure S1).

***Test 2.*** Across groups, exposure to a new smoking video led again to significantly increased craving (QSU-Brief: *F*_1,60_ = 12.25, *P* < 0.001; but not TCQ-SF: *P* > 0.05) and SC responses (*F*_1,60_ = 32.58, *P* < 0.001) and decreased HR (*F*_1,60_ = 10.67, *P* = 0.002) and BP responses (systolic BP: *F*_1,59_ = 4.54, *P* = 0.037; but not diastolic BP: *F*_1,60_ = 1.61, *P* > 0.05). For QSU-Brief scores, there was an interaction of stress condition by time (*F*_1,60_ = 5.82, *P* = 0.019; Figure S1E) driven by an increase in craving from pre- to post-video in stress group participants but not non-stress group participants (*P* < 0.001). An interaction of stress condition by time also emerged for systolic BP responses when controlling for baseline differences (*F*_1,59_ = 5.54, *P* = 0.022; Figure S1C), revealing greater decreases in BP in stress group (*P* < 0.002) than control participants.

***Test 3.*** Across groups, exposure to a new smoking video led to significantly increased SC (*F*_1,60_ = 27.21, *P* < 0.001) and decreased HR (*F*_1,60_ = 35.53, *P* < 0.001). There were no main effects on other measures and no group differences in craving or physiological responses (Figure S1).

***Across Baseline and Test Sessions.*** There was an interaction of session by time for SC (*F*_2.23,133.94_ = 13.66, *P* < 0.001) reflecting decreases in post-video SC from baseline to tests 1 (*P* < 0.001), 2 (*P* = 0.028) and 3 (*P* = 0.005). While there was no main effect on BP across sessions, there was a stress condition by time interaction (systolic BP: *F*_1,177_ = 4.77, *P* = 0.033, η_p_^2^ = 0.075) driven by within-session decreases in response to the smoking videos in the stress (*P* < 0.001) and control (*P* = 0.034) groups (Figure S1C). A time by session interaction was detected for diastolic BP (*F*_3,180_ = 5.38, *P* = 0.001), reflecting a significant decrease in responses to the smoking videos at test 1 (*P* < 0.001).

**Supplemental References**

1. Ghahremani DG, Faulkner P, Cox CM, London ED. Behavioral and neural markers of cigarette-craving regulation in young-adult smokers during abstinence and after smoking. Neuropsychopharmacology. 2018;43(7):1616-1622.

2. Tong C, Bovbjerg DH, Erblich J. Smoking-related videos for use in cue-induced craving paradigms. Addict Behav. 2007;32(12):3034-3044.

3. Gilbert D, Rabinovich N. International smoking image series (with neutral counterparts), version 1.2. Carbondale, Integrative Neuroscience Laboratory, Department of Psychology, Southern Illinois University. 1999.

4. Chanon VW, Sours CR, Boettiger CA. Attentional bias toward cigarette cues in active smokers. Psychopharmacology. 2010;212(3):309-320.

5. Mogg K, Field M, Bradley BP. Attentional and approach biases for smoking cues in smokers: An investigation of competing theoretical views of addiction. Psychopharmacology. 2005;180(2):333-341.

6. Luijten M, van Meel CS, Franken IH. Diminished error processing in smokers during smoking cue exposure. Pharmacol Biochem Behav. 2011;97(3):514-520.

7. Geier A, Pauli P, Mucha R. Appetitive nature of drug cues confirmed with physiological measures in a model using pictures of smoking. Psychopharmacology. 2000;150(3):283-291.

8. Mucha R, Geier A, Pauli P. Modulation of craving by cues having differential overlap with pharmacological effect: Evidence for cue approach in smokers and social drinkers. Psychopharmacology. 1999;147(3):306-313.

9. Mucha RF, Pauli P, Weber M, Winkler M. Smoking stimuli from the terminal phase of cigarette consumption may not be cues for smoking in healthy smokers. Psychopharmacology. 2008;201(1):81-95.

10. Dedovic K, Renwick R, Mahani NK, Engert V, Lupien SJ, Pruessner JC. The Montreal Imaging Stress Task: Using functional imaging to investigate the effects of perceiving and processing psychosocial stress in the human brain. J Psychiatry Neurosci. 2005;30(5):319-325.

11. Jones A, Steeden JA, Pruessner JC, Deanfield JE, Taylor AM, Muthurangu V. Detailed assessment of the hemodynamic response to psychosocial stress using real‐time MRI. J Magn Reson Imaging. 2011;33(2):448-454.

12. Brugnera A, Zarbo C, Tarvainen MP, Marchettini P, Adorni R, Compare A. Heart rate variability during acute psychosocial stress: A randomized cross-over trial of verbal and non-verbal laboratory stressors. Int J Psychophysiol. 2018;127:17-25.

13. Voellmin A, Winzeler K, Hug E, Wilhelm FH, Schaefer V, Gaab J, et al. Blunted endocrine and cardiovascular reactivity in young healthy women reporting a history of childhood adversity. Psychoneuroendocrinology. 2015;51:58-67.

14. Pollock V, Cho DW, Reker D, Volavka J. Profile of Mood States: The factors and their physiological correlates. J Nerv Ment Dis. 1979;167(10):612-614.

15. Woicik PA, Stewart SH, Pihl RO, Conrod PJ. The substance use risk profile scale: A scale measuring traits linked to reinforcement-specific substance use profiles. Addict Behav. 2009;34(12):1042-1055.

16. Donnellan MB, Oswald FL, Baird BM, Lucas RE. The mini-IPIP scales: Tiny-yet-effective measures of the Big Five factors of personality. Psychol Assess. 2006;18(2):192-203.

17. Cohen S, Williamson G. Perceived stress in a probability sample of the United States In: Spacapan S, Oskamp S, eds. The social psychology of health: Claremont Symposium on applied social psychology. Newbury Park, CA: Sage; 1988.

18. American Psychiatry Association. Diagnostic and Statistical Manual of Mental Disorders. 5 ed. Washington, DC: American Psychiatric Publishing; 2013.

19. Sealed Envelope Ltd. Create a blocked randomisation list. <https://www.sealedenvelope.com/simple-randomiser/v1/lists>. Accessed 2 February 2019.

**Supplementary Figures and Tables**

**Supplementary Figure 1. Cue reactivity assessment.** There were no differences between groups in terms of cue reactivity (**A**, **B**, **D**, **F**) with the exception of baseline differences in systolic BP (controlled for at test) and changes in systolic BP (**C**) and QSU-Brief (**E**) responsivity at test 2 in the stress group. BP: blood pressure; TCQ-SF: Tobacco Craving Questionnaire – Short Form; QSU-Brief: Questionnaire on Smoking Urges – Brief; bpm: beats per minute; mmHg: millimetre of mercury. EMM: estimated marginal mean; SEM: standard error of the mean. ** *P* < 0.01, ** *P* < 0.01, *** *P* < 0.001.

**Supplementary Figure 2. Systolic BP changes during the intervention correlated with decreased cigarette use.** Larger decreases in daily cigarette use at follow-up were associated with (**A**) higher systolic BP post-retrieval and (**B, C**) larger changes in systolic BP during phase 2 (extinction phase) across all groups. BP: blood pressure.

**Supplementary Figure 3. Physiological and craving responses in all four groups during the behavioral intervention.** During phase 1 (**A**), participants exposed to the stress task exhibited significant increases in SC, QSU-Brief and TCQ-SF scores. During phase 2 (**B**), physiological and craving measures did not significantly differ between groups, but there were significant main effects of time (pre- versus post-extinction phase) showing an increase in SC and decreases in HR and systolic BP across all groups combined. BP: blood pressure; HR: heart rate; SC: skin conductance; TCQ-SF: Tobacco Craving Questionnaire – Short Form; QSU-Brief: Questionnaire on Smoking Urges – Brief. EMM: estimated marginal mean; SEM: standard error of the mean.

* *P* < 0.05, ** *P* < 0.01, *** *P* < 0.001. Main effect of time (pre- to post-phase): ⟠ *P* < 0.05; ⟠⟠⟠ *P* < 0.001.

**Supplementary Figure 4.** **Change in cigarette use in all four groups.** (**A**) There was a significant decrease in cigarette use across sessions, collapsed across all groups. A Group by Session interaction was not obtained *(P* = 0.122), but planned comparisons identified significant decreases in cigarette use in each of the two stress groups (all *P*s < 0.01, from baseline to test 3) but not the control task groups. (**B**) A significant Group by Session interaction (*P* = 0.035) reflected greater percent changes in cigarette use in the Stress – Smoking Cue group at week 6 versus week 2 (*P =* 0.024), and trends for greater decreases in the Stress – Smoking Cue vs. the Control – Smoking Cue groups (*P* = 0.026, LSD) and Stress – Neutral Cue vs. Control – Smoking Cue groups (*P* = 0.074, LSD); these latter two effects plausibly contributed to the Group by Session interaction, but both were weakened further following Bonferroni correction (*P* = 0.16 and *P* = 0.44, respectively). There were no significant decreases in cigarette use in the Control – Smoking Cue group (all *P*s > 0.37) whereas cigarette use decreased from baseline to test session 2 (*P* = 0.010) and baseline to test session 3 (*P* = 0.005) in the Stress – Neutral Cue group. EMM: estimated marginal mean; SEM: standard error of the mean; LSD: Least Significant Difference test. * *P* ≤ 0.05, compared to same group at week 2. † *P* < 0.05 (LSD), compared to control task group. Main effect of session: ⟠ *P* < 0.05, ⟠⟠⟠ *P* < 0.001. Change in the Stress – Smoking Cue group: § *P* < 0.05*,* Change in the Stress – Neutral Cue group: ¤ *P* < 0.05.

**Supplementary Figure 5. Cue reactivity assessment for all four groups.** There were no differences between the four groups in terms of cue reactivity (**A**, **B**, **D**, **E, F**) with the exception of systolic BP at baseline (**C**). Although the groups were not yet treated differently at baseline, there was a significant decrease in systolic BP the Control-Neutral Cues (*P* = 0.008). BP: blood pressure; TCQ-SF: Tobacco Craving Questionnaire – Short Form; QSU-Brief: Questionnaire on Smoking Urges – Brief; bpm: beats per minute; mmHg: millimetre of mercury. EMM: estimated marginal mean; SEM: standard error of the mean. * *P* < 0.05, ** *P* < 0.01. Main effect of time (pre- to post-cue): ⟠ *P* < 0.05, ⟠⟠ *P* < 0.01, ⟠⟠⟠ *P* < 0.001.

| **Supplementary Table 1. Mean Craving, Cigarette Dependence and Motivation to Quit Smoking Scores Across Sessions** | | | | | | | |
| --- | --- | --- | --- | --- | --- | --- | --- |
| **Measure** | **Group** | **Session** | | | | **Statistics** | |
|  |  | **Baseline** | **Test 1** | **Test 2** | **Test 3** | **Across Sessions (*F* value)** | **Stress Condition x Session (*F* value)** |
| CWS-21 - Craving | Control | 3.11 (0.18) | 2.66. (0.17) | 2.49 (0.20) | 2.43 (0.17) | 7.52*** | 0.76 |
|  | Stress | 3.02 (0.18) | 2.88 (0.17) | 2.50 (0.19) | 2.68 (0.16) |  |  |
| FTCD | Control | 5.97 (0.21) | - | 5.56 (0.33) | 5.18 (0.37) | 10.77*** | 0.45 |
|  | Stress | 6.13 (0.22) | - | 5.53 (0.35) | 4.93 (0.38) |  |  |
| Contemplation Ladder | Control | 4.94 (0.29) | 5.28 (0.28) | 5.75 (0.29) | 6.03 (0.27) | 9.98*** | 0.92 |
|  | Stress | 5.00 (0.30) | 5.30 (0.29) | 5.40 (0.30) | 5.67 (0.28) |  |  |

CWS-21: Cigarette Withdrawal Scale – 21; FTCD: Fagerström Test for Cigarette Dependence. *** *P* < 0.001.
